# Higher-order mode supercontinuum generation in dispersion-engineered liquid-core fibers

**DOI:** 10.1038/s41598-021-84397-1

**Published:** 2021-03-05

**Authors:** Ramona Scheibinger, Niklas M. Lüpken, Mario Chemnitz, Kay Schaarschmidt, Jens Kobelke, Carsten Fallnich, Markus A. Schmidt

**Affiliations:** 1 Leibniz Institute of Photonic Technology, Albert-Einstein-Str. 9, 07745 Jena, Germany; 2 Institute of Applied Physics, University of Münster, Corrensstraße 2, 48149 Münster, Germany; 3 INRS-EMT, 1650 Boulevard Lionel-Boulet, Varennes, QC J3X 1S2 Canada; 4 MESA+ Institute for Nanotechnology, University of Twente, 7500 AE Enschede, The Netherlands; 5 Otto Schott Institute of Material Research, Friedrich Schiller University Jena, Fraunhoferstrasse 6, 07743 Jena, Germany

**Keywords:** Fibre optics and optical communications, Nonlinear optics, Solitons, Supercontinuum generation

## Abstract

Supercontinuum generation enabled a series of key technologies such as frequency comb sources, ultrashort pulse sources in the ultraviolet or the mid-infrared, as well as broadband light sources for spectroscopic methods in biophotonics. Recent advances utilizing higher-order modes have shown the potential to boost both bandwidth and modal output distribution of supercontinuum sources. However, the strive towards a breakthrough technology is hampered by the limited control over the intra- and intermodal nonlinear processes in the highly multi-modal silica fibers commonly used. Here, we investigate the ultrafast nonlinear dynamics of soliton-based supercontinuum generation and the associated mode coupling within the first three lowest-order modes of accurately dispersion-engineered liquid-core fibers. By measuring the energy-spectral evolutions and the spatial distributions of the various generated spectral features polarization-resolved, soliton fission and dispersive wave formation are identified as the origins of the nonlinear broadening. Measured results are confirmed by nonlinear simulations taking advantage of the accurate modeling capabilities of the ideal step-index geometry of our liquid-core platform. While operating in the telecommunications domain, our study allows further advances in nonlinear switching in emerging higher-order mode fiber networks as well as novel insights into the sophisticated nonlinear dynamics and broadband light generation in pre-selected polarization states.

## Introduction

Supercontinuum generation (SCG) represents a highly efficient spectral broadening mechanism that allows distributing electromagnetic energy of an ultrashort pulse across defined spectral domains^1^. Via dispersion management and strong light confinement, fiber-based SCG led to both the observation of novel nonlinear physics (e.g., soliton fission^2^, soliton self-frequency shift^3^, nonlinear mode coupling^4,5^), as well as to applications in multiple fields (e.g., tomography^6^, spectromicroscopy^7^, hyperspectral LiDAR^8^). The majority of studies focused on nonlinear frequency conversion in the fundamental fiber mode, which can show limitations regarding dispersion tunability, variability in output mode profile and spectral broadening.

Recent studies extended this state-of-the-art by employing higher-order modes (HOMs) which provide manifold sophisticated dispersion landscapes and allow the observation of new nonlinear phenomena on the basis of nonlinear interaction of different waveguide modes. Effects such as intermodal four-wave mixing^4,5^ and intermodal Raman scattering^9,10^ enhance the spectral broadening through energy transfer between different modes while spatial mode control at the fiber input offers a new degree of freedom^11–13^, which results in, e.g., accelerated nonlinear interactions observed in tapered multimode fibers^14^. Many of those studies are focused on highly multimode waveguide systems supporting hundreds to thousands of modes, the nonlinear dynamics of which, notably, can be described thermodynamically^15^. However, the characteristics of the coupling between individual modes remain largely uncovered. HOMs have dispersion landscapes and spatial field profiles that are fundamentally different to their fundamental counterpart, allowing for tailoring the light generation process and thus being principally highly relevant for many applications such as sub-diffraction limited focusing^16^ or super-resolution imaging^17^. Readily applicable broadband laser sources for such applications require a detailed understanding of the nonlinear dynamics within waveguides, which support a comparably low number of modes.

Controlling nonlinear interaction of HOMs in multimode fibers is challenging, since, in addition to temporal effects, nonlinear intermodal coupling dynamics significantly influence the spectral broadening processes. In particular, disentangling and steering individual mode contributions to nonlinear conversion processes in conventional few-mode glass fibers is hampered by variations in (1) the modal dispersion over length due to imperfections of the drawing process, (2) the mode composition and mode distinguishability due to variations in the gradual refractive index transitions between core and cladding, and (3) the mode stability due to material stresses imposed by environmental changes. Nonlinear experiments with higher-order modes (mainly the LP_02_ mode) were conducted in PCFs^18,19^, and solid, silica based HOM fibers^20,21^ with ring-shaped index profiles favoring the guidance of specific higher-order modes, and lead to an extension of the wavelength region of anomalous dispersion (AD).

**Figure 1:**
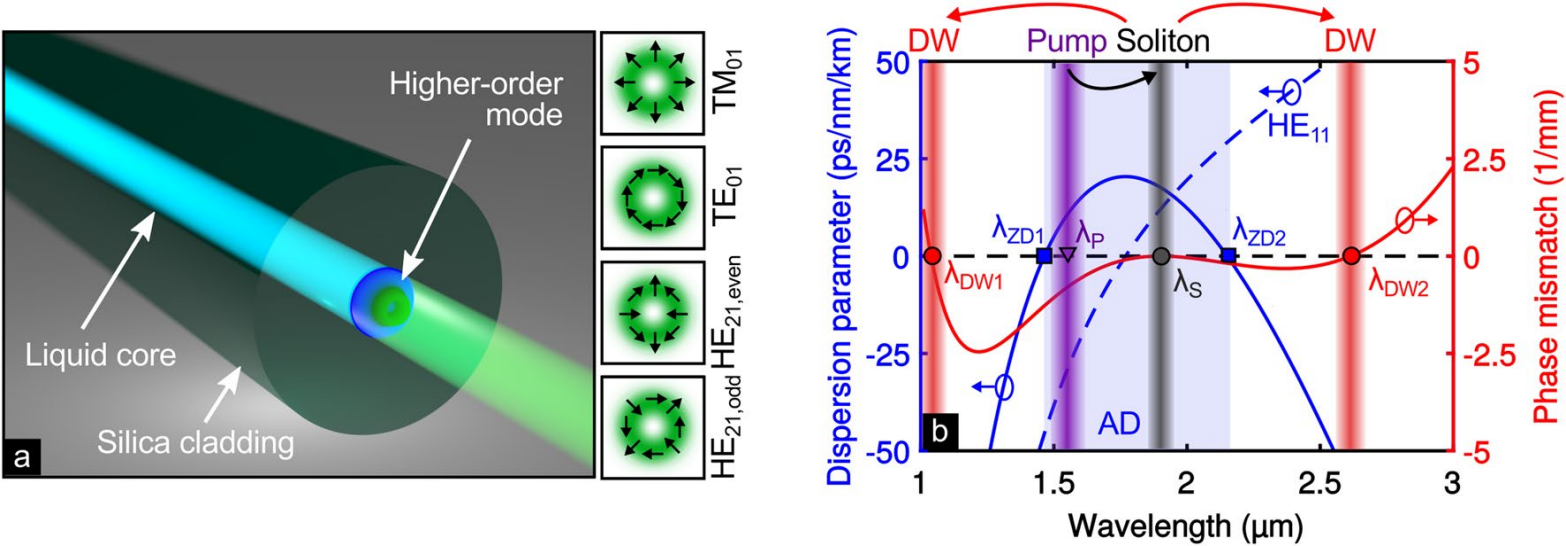
Double zero-dispersion wavelengths for HOMs in LCFs. (**a**) Sketch of higher-order mode propagation in a liquid core fiber, with polarization and intensity distributions of the investigated TM_01_, TE_01_, and HE_21_ modes shown on the right (black arrows indicate the direction of the electric field at a fixed point in time). (**b**) Schematic of dual dispersive wave (DW) generation by soliton fission for the TM_01_ mode in a CS_2_-core silica fiber (Ø_core_ = 4 µm). The group velocity dispersion profile (blue line) has two ZDWs (λ_ZD1_ and λ_ZD2_) and an anomalous dispersive regime (AD, blue shaded) at the pump wavelength λ_P_ = 1.56 µm (purple bar), which allows to generate a soliton (gray bar) with an exemplary wavelength of λ_S_ = 1.9 µm. Enclosed by two ZDWs, the soliton transfers energy to two DWs (red bars), whose wavelengths (λ_DW1_ and λ_DW2_) can be determined by the phase-matching condition (red line). For the same fiber geometry, the fundamental mode (blue dashed line) is normal dispersive at λ_P_.

In the present work, we demonstrate few-mode step-index liquid-core fibers (LCFs), with precisely adapted circular core diameters, as a novel platform to study nonlinear dynamics in HOMs. LCFs benefit from (1) distinct step-index refractive index profiles with index contrasts comparable to soft-glass fibers^22^, enabling straightforward mode modeling and large effective index differences between different modes, (2) the lack of mode perturbations through internal material stress, and (3) potential local control over the nonlinear dynamics via temperature^23^. Those benefits clearly distinguish the capabilities of LCFs from other nonlinear fibers, and allow to excite different types of HOMs (TE_0n_, TM_0n_, HE_1n_, HE_n1_, etc.) separately with high efficiency in one and the same fiber, when modifying the incoupled beam profile and its polarization accordingly. To highlight those capabilities, we study nonlinear frequency conversion in three HOMs (TM_01_, TE_01_ and HE_21_, shown in Fig. 1a) in dispersion-managed LCFs experimentally and numerically in the context of SCG. In contrast to the fundamental mode of most step-index fibers, the individual HOMs in our system feature anomalous dispersion at 1.56 µm and two zero-dispersion wavelengths (ZDWs) in the near-infrared at practically well-accessible core diameters of around 4 µm. Pumping with ultrashort pulses at telecommunication wavelengths leads to mode-selectively soliton-driven SCG via dual dispersive wave generation. Specifically, we analyze the spectral power distribution between the three key spectral features—pump, soliton and dispersive wave—for various configurations and we discover a strong dependence of the light generation process on the fiber core diameter and on the exciting input mode. This increased sensitivity of the HOMs allows for an increased tunability of the output frequency spectrum.

## Methods

### Higher-order modes in liquid-core fibers

In the context of this work, carbon disulfide (CS_2_) filled fiber-type silica capillaries (i.e., LCFs) are used, which are suitable for near-infrared applications due to their wide transparency range up to 3.2 µm wavelength^24–26^, their high nonlinear refractive index (0.7 × 10^–19^ m^2^/W)^27–29^ at pump wavelength λ_P_ = 1.56 µm, and their high linear refractive index (1.59 at λ_P_) allowing for tight mode confinement in combination with a silica cladding^30^.

In order to exploit higher-order modes in LCFs, the waveguide parameter *V* must exceed the single mode criterion of *V* > 2.405, which is fulfilled with core diameters Ø_core_ > 3.4 µm. For instance, a CS_2_-filled LCF with Ø_core_ = 3.9 µm (*V* = 5.2) supports eight modes at λ_p_, counting degenerated modes only once. Among all HOMs the TM_01_, TE_01_ and HE_21_ modes have the highest effective refractive indices, indicating that they are guided most robustly. Their effective refractive indices differ in the range of 10^–3^ for wavelengths around λ_P_ (see Fig. S1 in supplementary information I), such that random refractive index perturbations of the fiber do not cause strong linear coupling of the modes^31^. At λ = 2.1 µm the effective refractive indices of the TM_01_ and HE_21_ modes match, leading to direct linear coupling between them in case the corresponding spectral components reach this wavelength, which, however, is barely the case in our experiments. All three modes show ring-shaped intensity distributions, while the TE_01_ and TM_01_ modes are clearly distinguishable by their radial or azimuthal polarization (Fig. 1a). The two degenerated even and odd HE_21_ modes, in contrast, feature strongly varying polarization distributions along the azimuthal direction. In case of a small perturbation of the cylindrical symmetry, e.g., for slightly elliptical fibers, the degeneracy of both HE_21_ modes is lifted resulting in slightly different propagation constants.

### Soliton fission based supercontinuum and phase-matching condition of dispersive waves

Soliton-driven SCG processes rely on the formation of higher-order solitons, which require anomalous dispersion. For this purpose, the dispersion parameter *D* = − *λ / c* × *d*^*2*^*n*_*eff  *_*/ dλ*^*2*^, calculated from the effective refractive index *n*_*eff*_ = *λ / 2π* × *β*, has to be positive at λ_P_ (anomalous dispersion). Here, *β* is the propagation constant of the respective mode. In the investigated LCFs the TM_01_, TE_01_ and HE_21_ modes show anomalous group velocity dispersion (GVD) at the pump wavelength λ_P_ = 1.56 µm. As an example, *D* of the TM_01_ mode (Ø_core_ = 4 µm) is depicted as a solid blue curve in Fig. 1b, showing an AD regime ((*D* > 0, blue shaded) around λ_P_ enclosed by two ZDWs (blue squares, labeled as λ_ZD_).

The main spectral broadening of soliton-driven SCG is obtained by the fission of higher-order solitons into their fundamental counterparts, being associated with the emission of excess energy to phase-matched linear radiation, i.e., to so-called dispersive waves (DW)s^2^. By self-phase modulation in combination with anomalous group velocity dispersion a higher-order soliton is formed, which is characterized by the soliton number *N* = (*L*_D_ /*L*_NL_)^1/2^ with the dispersive and nonlinear length *L*_D_ and *L*_NL_, respectively. Furthermore, *N* describes the number of fundamental solitons which are ejected from the higher-order soliton after fission^1^. The first released fundamental soliton is the most dominant and transfers energy via intra-modal phase-matching to DWs, whose central wavelengths (*λ*_DW_) typically lie in the normal dispersion (ND) regime and can be calculated by the corresponding intra-modal phase-matching condition

1 $$\beta \left( {\lambda_{DW} } \right) - \beta \left( {\lambda_{S} } \right) + 2\pi c\left( {\lambda_{S}^{ - 1} - \lambda_{DW}^{ - 1} } \right) \times \beta_{1} \left( {\lambda_{S} } \right) = \frac{1}{2}\gamma P_{S} ,$$

with the propagation constants *β* at *λ*_DW_ and soliton wavelength *λ*_S_, and the inverse group velocity *β*_1_ = *dβ */ *dω* =  -*  λ*^2^ /* 2πc* × *dβ */* dλ* at *λ*_S_^32^. The additional nonlinear phase (RHS of Eq. 1) depends on the nonlinear parameter *γ* and the peak power of the first fundamental soliton *P*_S _= *P*_peak_ × (2* N − *1)^2 ^/ *N*^2^, calculated from the injected peak power *P*_peak_ and the soliton number *N*.

Assuming the first fundamental soliton being generated at 1.9 μm (gray background, far away from the pump for better visibility) the phase-matching condition (red curve) suggests energy transfer to two DWs (red backgrounds) located in the ND regime on either side of λ_P_. While the short-wavelength DW might be already generated during the initial fission process due to spectral overlap with the pump^33^, we assume that the long-wavelength DW is generated due to spectral overlap with the ejected fundamental soliton after the fission process (for details see phase-matching calculations for different soliton wavelengths in Fig. S2 of the supplementary information II). We suppose the following underlying mechanism: towards the second ZDW the group velocity dispersion decreases, forcing the soliton to temporally narrow to maintain N = 1, i.e., to broaden spectrally. Close to the second ZDW, this soliton self-compression eventually reaches the correct spectral overlap of the fundamental soliton with the phase-matched DW2. Consequently, the efficiency of the energy transfer to the long-wavelength dispersive wave depends on the split-off soliton and not on the pump (also considering a non-negligible group delay between pump and first soliton by the time of DW2 generation). This underpins soliton fission in double-ZDW dispersion settings as a highly attractive scheme for coherent nonlinear frequency conversion over broad bandwidths^34–36^. The three HOMs have slightly different GVDs and ZDWs, such that the wavelengths of generated solitons and DWs are expected to vary between the modes. In contrast to the three HOMs, the fundamental mode (HE_11_, dashed blue line in Fig. 1b) has only one ZDW at 1.8 μm and is normal dispersive (D < 0) at λ_P_, discarding it for soliton-driven SCG in the telecom regime. Achieving double-ZDW dispersion landscapes for the fundamental mode of step-index waveguides usually requires sub-wavelength core diameters, making coupling and light guidance challenging^34–37^. Examples include experiments conducted in, e.g., a 8 µm chalcogenide fiber (pumped in the normal dispersive regime)^9^, and in micro-structured fibers focusing on the fundamental mode only^38–40^.

### Experimental setup and simulations

In the experiments, CS_2_-based LCFs of 8.5 cm length and with different core diameters are implemented by mounting empty fiber-type silica capillaries (available inner diameters Ø_core_ = 3.5 µm, 3.9 µm, 4.4 µm) in-between two optofluidic holders, which include small liquid reservoirs that were filled with CS_2_ via capillary forces and closed by sapphire or silica windows^30^. In the following, all measurements are performed with the LCFs with Ø_core_ = 3.9 µm, while the two additional CS_2_-core fibers (Ø_core_ = 3.5 µm, 4.4 µm) are used to study the dependence on the core diameter.

**Figure 2:**
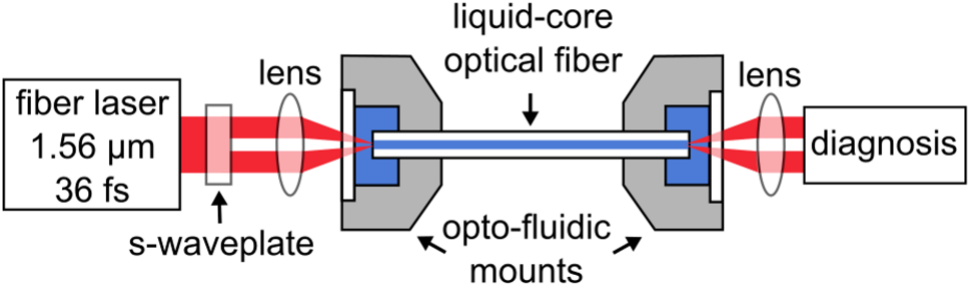
Experimental setup for soliton-driven HOM-SCG in LCFs. The ultrashort pulses are spatially modified by a commercial s-waveplate and coupled into the fiber by an aspheric lens (*NA* = 0.55, *f* = 4.51 mm). For the outcoupling another aspheric lens (*NA* = 0.56, *f* = 6.00 mm) is used to feed the light into a large core fluoride fiber connected to a spectrometer or to image the beam with an infrared camera.

The experimental setup for HOM-SCG consists of an ultrafast fiber laser (Toptica FemtoFiber pro IR, central wavelength 1560 nm, repetition rate 80 MHz, pulse duration 36 fs), an s-waveplate, the LCF sample, as well as in- and out-coupling lenses and diagnostics (Fig. 2). The s-waveplate (Altechna) creates the desired input polarization state in order to convert the linear polarized Gaussian input beam to a ring-shaped beam that resembles either the TM_01_ or TE_01_ fiber mode^41^. To excite the HE_21_ mode a half-wave plate is added after the s-waveplate. The spatial distributions of the Stokes parameters^42^ are measured before the coupling lens to verify that beam profile and polarization distribution match the targeted HOM (see supplementary information III, Fig. S3). The Stokes parameters S_1_ and S_2_, which characterize the orientation of linear polarization and are substantial for distinguishing TM_01_, TE_01_ and HE_21_ modes, are in good agreement with the theoretical fiber modes. The intensity distribution (S_0_) shows a clear ring shape, whereas, in contrast to the ideal fiber modes, the circular polarization (S_3_) does not vanish completely. Due to losses of the quarter-wave plate, which was only inserted to measure the S_3_ parameters, the subsequent discussion of the fiber output modes focuses exclusively on the first three Stokes parameters. The estimated coupling efficiency to the higher-order modes is about 34%, corrected for reflection losses at the interfaces of lenses and windows and absorption in the fiber.

In order to study the energy-spectral evolution of the SCG process, the injected pulse energy is increased stepwise for each of the three higher-order input modes separately. The spectra were measured by two spectrometers with different spectral ranges and noise levels, and combined at a wavelength of approx. 1.7 µm using an offset correction. Note that the noise level values at the long wavelength side have been artificially reduced to the noise level at short wavelengths to avoid misinterpretations. For each mode the measured spectra are normalized to the maximum spectral power density of all spectra, allowing for the direct comparison of the evolutions. The modal contents of the output spectra are investigated via determining the Stokes parameters in the three spectral domains of pump, soliton and DW using appropriate spectral filters. The strong sensitivity of the SCG process on core diameter is studied by comparing spectral measurements for TE_01_ input mode in the three different LCFs (Ø_core_ = 3.5 µm, 3.9 µm, 4.4 µm).

The experiments are supported by simulations of the nonlinear pulse dynamics based on solving the multimode generalized nonlinear Schrödinger Equation^36^

2 $$\begin{aligned} \frac{{\partial A_{p} }}{\partial z} & = i\left( {\beta_{0}^{\left( p \right)} - \beta_{0} } \right)A_{p} - \left( {\beta_{1}^{\left( p \right)} - \beta_{1} } \right)\frac{{\partial A_{p} }}{\partial t} + i \mathop \sum \limits_{n \ge 2} \frac{{\beta_{n}^{\left( p \right)} }}{n!}\left( {i\frac{\partial }{\partial t}} \right)^{n} A_{p} \\ & \quad + i \frac{{n_{2} \omega_{0} }}{c}\left( {1 + i\tau_{0} \frac{\partial }{\partial t}} \right) \mathop \sum \limits_{l,m,n} \left\{ {Q_{plmn } \left( {\omega_{0} } \right)\left[ {2\left( {1 - f_{R} } \right)A_{l} A_{m} A_{n}^{*} + 3f_{R} A_{l} \smallint d\tau h\left( \tau \right)A_{m} \left( {t - \tau } \right)A_{n}^{*} \left( {t - \tau } \right)} \right]} \right\}, \\ \end{aligned}$$

as introduced by Poletti and Horak^5^, where *A*_*p*_ denotes the amplitude of mode *p*, *z* the propagation distance, *β*_*k*_^*(p)*^ the *k*-th dispersion coefficient of mode *p*, *β*_0_ an overall phase factor, 1/* β*_1_ the velocity of a reference frame, *t* the time in the reference frame, *n*_2_ the nonlinear refractive index coefficient, *ω*_0_ the angular center frequency, *τ*_0_ the shock time constant, *Q*_*plmn*_ the nonlinear coupling coefficients, *f*_*R*_ the fractional contribution of the non-instantaneous response, and *h(τ)* the delayed response function. The non-instantaneous response of CS_2_ consists of a rotational response and a vibrational response^24^. The ultrafast rotational response of the liquid’s molecules is negligible here, as the molecular fraction f_R_ = 0.18 calculated for 36 fs is sufficiently small^27,28,43^. A significant impact of this type of nonlinear response, which is not present in case of solid materials, requires an f_R_ of 0.7 or higher for fiber lengths of around 10 centimeters^30^. In contrast, the vibrational response shows a narrow band peak at 19.7 THz^44,45^, while the amplitude of the response has not been quantified to an extent that it can be used in nonlinear pulse propagation simulations. Preliminary simulations qualitatively revealed that the most dominant Raman peak at 19.7 THz only induces slight changes of the generated supercontinua (e.g., an additional weak short-wavelength DW or a step-wise red-shift of the soliton with increasing pulse energy), whereas all major spectral features (e.g., spectral location of the short-wavelength DW and soliton) remain unchanged, giving us reason to neglect the Raman response in all simulations presented in this work. The propagation loss was neglected due to the short fiber lengths used in the experiments. A measured spectrum of the pump laser with pulse duration of 36 fs is used as the input pulse and shot-noise was included by adding half of the photon energy with a random phase to each frequency mode^46^. Each of the depicted simulated spectra at the fiber output is the sum of the power spectral densities of all contributing modes. The averaging of one hundred simulated output spectra that include random shot-noise (following Dudley and Cohen^47^) revealed an extremely high first-order degree of coherence > 99.9% covering almost the entire generated bandwidth, see supplementary information V, Fig. S4.

The power distribution of the HOM-inputs for the simulation (i.e., the different modal amplitudes) is estimated by correlating the theoretical fiber modes^48^ with the electrical fields at the location of the LCF after focusing^49^. For this purpose, the electric fields before the coupling lens are extracted from the measured intensity profiles used for the Stokes parameter analysis, and the electric field distributions in the focus are calculated following reference^50^ (see supplementary information IV for details). The overlap calculations show that in all cases the injected beam profiles have the strongest overlap with the desired fiber mode. Specifically, the HE_21_-like beam matches the theoretical fiber mode by more than 99.7% and is thus assumed to be solely excited. In case of the TM_01_ and TE_01_ modes, however, the contributions in the other modes are non-negligible (in the order of several percent) and, therefore, the input powers are distributed among the different HOMs accordingly. All simulations assume a perfectly cylindrical LCF geometry (Ø_core_ = 3.9 µm).

## Results

### SCG with selective HOM excitation

**Figure 3:**
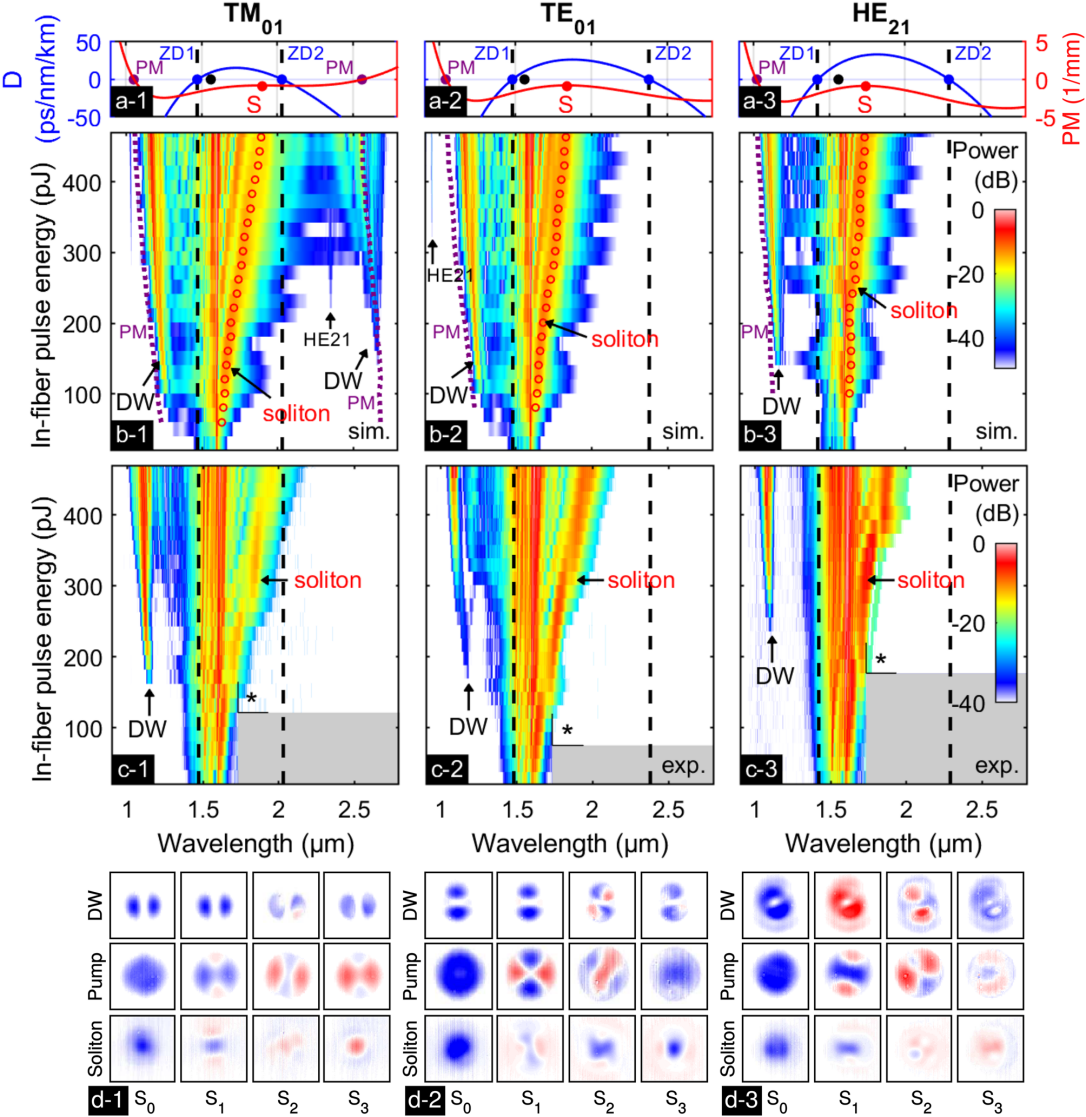
Supercontinuum generation inside a CS_2_-LCF (core diameter Ø_core_ = 3.9 µm) in case of TM_01_- (column 1), TE_01_- (column 2) and HE_21_-like excitation (column 3). Row (**a**): spectral distribution of the group velocity dispersion parameter *D*, with ZDWs (ZD1 and ZD2) marked as black dashed lines. The pump wavelength λ_P_ is shown as black dot. The phase-mismatch (PM) is given in red (right axis) for the highest in-fiber energy (483pJ) using the corresponding soliton wavelength S and Eq. (1). The resulting phase-matched wavelengths are marked with PM. Row (**b**): corresponding normalized nonlinear pulse evolution simulations including intermodal coupling. The energy-dependent phase-matching wavelengths (purple dotted lines) are calculated for the respective soliton wavelengths (red circles). The input power distribution across the different modes is as follows: (**b-1**) 99% TM_01_, 1% HE_21_, (**b-2**) 96% TE_01_, 3% TM_01_, 1% HE_21_, (**b-3**) 100% HE_21_. The plots show the accumulated power in all simultaneously injected modes. Row (**c**): measured normalized spectral evolutions for increasing in-fiber pulse energy. The asterisk (*) marks the start of the spectra with reduced dynamic range, detected with a less sensitive spectrometer, whose noise floor was reduced artificially. No experimental data is available in the gray shaded regions. The colorbar at the right corresponds to all measured spectra. Row (**d**): measured spatial distribution of the Stokes parameters at an input pulse energy of ≈ 400 pJ in the spectral intervals of the three main spectral features (top row: dispersive wave (DW), center row: pump, bottom row: soliton.) The blue (red) color indicates a value of 1 (−1).

First, SCG measurements were performed to investigate the spectral evolution dependent on the excited HOM by measuring the energy-spectral evolutions in case each of the targeted modes (TM_01_, TE_01_ and HE_21_) is excited as purely as possible in the CS_2_-LCF (Ø_core_ = 3.9 µm, L = 8.5 cm) and placing the results in context of multimode simulations, see Fig. 3. All spectra show a red-shifting soliton and the generation of a DW. Note that the soliton red-shift in the spectral/energy evolutions does not originate from Raman effects but from increasing input pulse energies, e.g., soliton recoil^40,51^, which can also be seen from the exemplary simulations along fiber length presented for the TM_01_ case in supplementary information V Fig. S5. The evolutions with pulse energy of the TM_01_ and TE_01_ modes show strong similarities (Fig. 3c-1,c-2), especially regarding wavelength and pulse energy at the onset of the DWs (TM: 1.14 µm /162 pJ, TE: 1.17 µm /169 pJ) and the similar DWs’ wavelengths at a constant energy (see Table 1), which results from the almost identical ZDWs (TM_01_: 1.47 µm, TE_01_: 1.48 µm) and dispersion coefficients *D* (TM_01_: 9.3 ps /nm /km, TE_01_: 10.8 ps /nm /km) at λ_P_ (Fig. 3a). In contrast, the HE_21_ mode has its first ZDW at shorter wavelength (1.42 µm) resulting in a shorter wavelength of the DW (1.10 µm (Fig. 3c-3)). However, an approximately doubled dispersion coefficient *D* (20.6 ps /nm /km) at λ_P_ matches with the DW to appear at significantly higher pulse energy (236 pJ). A consequence of the larger dispersion parameter and the higher onset energy visible in the experiments is the weaker soliton red-shift at maximum pulse energy (470 pJ) for HE_21_-excitation (1.89 µm) compared to the TM and TE situations (1.97 µm and 1.96 µm)^32^. Note that the soliton number at the soliton wavelength is very similar for all three modes, see Table 1. The simulated spectra (Fig. 3b) show all mentioned spectral features and confirm the higher onset fission energy and the reduced final bandwidth of the HE_21_ case. The different strengths of DWs, pump and soliton in experiments and simulations can be related to chromatic aberrations when coupling to the spectrometer at the out-coupling side and favoring distinct wavelength regions, while suppressing others.

**Table 1:** Comparison of soliton and DW wavelengths.

mode	λ_ZD1_ [µm]	λ_ZD2_ [µm]	λ_S_ [µm]	λ_DW1_ (PM) [µm]	λ_DW1_ (sim) [µm]	λ_DW1_ (exp) [µm]	λ_DW2_ (PM) [µm]	λ_DW2_ (sim) [µm]	N
TM_01_	1.47	2.03	1.85	1.05	1.17	1.10	2.57	2.56	3
TE_01_	1.48	2.37	1.80	1.06	1.18	1.09	3.35	3.40	2
HE_21_	1.42	2.29	1.71	1.04	1.11	1.09	3.21	3.23	2

The wavelengths of DWs are obtained by phase-matching calculations (PM), nonlinear simulations (sim) and experiments (exp) for TM_01_, TE_01_ and HE_21_ mode in a 3.9 µm CS_2_-core fiber at 400 pJ. The soliton number N is calculated for the indicated soliton wavelength λ_S_.

In comparison to experiments, the simulated DWs are generated at approximately 100 pJ lower energies and are located at 50nm to 100 nm longer wavelengths for all modes. Due to the limited contrast of the used spectrometers (40 dB for wavelengths < 1.7 µm), the experimental DWs might be already present but undetectable for lower input powers. Based on the same reason, the long-wavelength DW, located around 2.6 µm in the TM_01_ mode as suggested by simulations, could not be detected in experiments. Its power of approx. − 25 dB to − 40 dB lies below the noise level of − 25 dB of the spectrometer used to characterize the long-wavelength features. Figure 3b shows that the spectral locations of the long-wavelength DWs are in good agreement with the calculations of the phase-mismatch (purple dotted line) calculated by Eq. (1), for direct comparison see Table 1. The second ZDW of the TM_01_ mode is much closer to the pump than in case of the TE_01_ and HE_21_ modes, thus explaining why the long-wavelength dispersive wave lies at 2.56 µm for the TM_01_ mode, and beyond the displayed region for the other modes (TE_01_: 3.33 µm, HE_21_: 3.20 µm, see supplementary information V Fig. S6). For the short-wavelength DWs the phase-matching wavelengths (purple dotted lines in Fig. 3b) show a similar shift with increasing energy like the simulated DWs while being located at slightly shorter wavelengths. The spectral broadening of the long-wavelength DW in Fig. 3b-1 results from a stronger spectral overlap with the soliton, which is closer to the long-wavelength DW for higher in-fiber energies. Note that this effect has been previously observed in other publications^34,38,40^. This good agreement between phase-matching calculations and nonlinear simulations justifies the assumptions of the simulations, e.g. that the Raman response was neglected. The minor differences between experiments and simulations may result from uncertainties in the input coupling efficiencies. The deviations of the central wavelengths of the DWs originates from small uncertainties in the refractive index model of CS_2_, having a larger impact on HOMs than on the fundamental mode. The influence by fiber ellipticity, which would change the dispersion of the HOMs, was excluded by measuring SEM images of the fiber capillaries (ellipticity of the fiber core < 50 nm).

The power distribution among the different HOMs at the input, calculated from the input fields (i.e., in case of TE and TM excitation), leads to non-intuitive soliton dynamics visible in simulations: according to the modal overlap calculations a TM polarized input beam leads to a power fraction of 99% in the TM_01_ mode, while the remaining 1% is coupled to the HE_21_ mode. As shown in Fig. 3b-1, this has the consequence that the generation of the DW of the TM_01_ mode at 2.67 µm is accompanied by the simultaneous generation of a DW in the HE_21_ mode at 2.33 µm. Because the power of the weak HE_21_ mode’s DW does not exceed − 40 dB, it cannot be detected in experiments. As only 1% of the input power is injected into the HE_21_ mode, the corresponding peak power is not sufficient to enable soliton fission by its own, as the estimated soliton number of the HE_21_ mode is only *N* = 0.4. However, our simulations indicate that the fission within the stronger TM_01_ mode induces DW generation in the weaker HE_21_ mode via nonlinear intermodal coupling due to cross-phase modulation (XPM). The simulated temporal and spectral evolutions of the supercontinuum generation process as a function of position inside the fiber (shown in the supplementary information V Fig. S5) confirm that the generation of the DW of the HE_21_ mode occurs without a soliton being generated in this weak mode, but it rather is induced by the soliton fission of the strong TM_01_ mode, which happens simultaneously. Simultaneous DW generation also appears on the short-wavelength side, where the strong TM_01_ peak at 1.30 µm is superimposed by a weaker peak of the HE_21_ mode at 1.33 µm (see Fig. S6a in supplementary information V). While the HE_21_ mode was excited purely and did not show intermodal effects, the TE_01_ mode shows similar nonlinear interactions: the spectra of the weakly excited TM_01_ and HE_21_ modes (3% and 1%) do not show considerable broadening, while the individual spectra reveal new DW-related spectral components (TM_01_: 0.91 µm and 2.98 µm; HE_21_: 0.95 µm and 3.42 µm) induced by the TE_01_ mode (see Fig. S6b in supplementary information V). In contrast to the already known four-wave mixing-induced ‘intermodal Cherenkov radiation’^52–55^, further simulations verified that in our case the new spectral components are induced by intermodal XPM. Intermodal XPM was already reported by Essiambre et al. in a km-long fiber^56^, but to the best of our knowledge XPM-induced intermodal DW generation was not investigated yet. As the complex polarization profiles of the modes hampers the modal demodulation of the output spectra, the effect will be investigated in detail in a future study.

### Measured beam profiles of pump, soliton, and DW

To estimate the modal content of the prominent spectral features, the mode profiles in the spectral intervals of pump, soliton, and DW are determined for an input energy of approx. 400 pJ via the Stokes parameter analysis (see Fig. 3d). At λ_P_ the polarization distributions of all three modes (central row in Fig. 3d) resemble the theoretic distribution of the mainly excited HOM (see Fig. S3b in supplementary information III), confirming the correct excitation conditions.

Within the spectral window of the short-wavelength DWs (1.1 µm < λ < 1.2 µm, top row in Fig. 3d) dumbbell-shaped profiles with modified polarization distributions (see S_1_ and S_2_) were measured for all modes, which might be caused by small perturbations in the fiber or additional mode coupling effects not included in the simulations. In contrast to linearly polarized LP_11_ modes, which are linear combinations of TM_01_ or TE_01_ modes with HE_21_ modes^57^, the measured polarization distributions are not entirely unidirectional but include radial/azimuthal contributions. Furthermore, as only the TM_01_ mode simulation shows additional spectral power densities for the HE_21_ mode in this wavelength domain, the origin of the measured LP-like mode patterns cannot be explained by such a linear mode combination. Additional modal calculations show that LCFs with slightly elliptical cores can support linear dumbbell-shaped modes with a relatively linear polarization pattern, while the simulation indicates that this applies across the entire spectral domain investigated here, rather than for the short-wavelength side only. Therefore, our hypothesis is that the observed dumbbell-shaped profiles of the DW result from additional linear or nonlinear mode coupling effects.

Interestingly, the Stokes parameters for the solitons do not match any of the three HOMs or mixtures of those with other higher-order modes (bottom row, Fig. 3d). The spatial distributions of the Stokes parameters S_1_ and S_2_ qualitatively resemble those of the input mode with non-vanishing contributions in the center of the beam, where, however, the circular parameters S_3_ are comparably strong. We ascribe these measured polarization state of the solitons to imaging errors in this particular spectral domain, where the used camera has very low sensitivity.

For all three excitation configurations a consistent modal behavior is measured in the three spectral domains. In case of the degenerate HE_21_ mode, a 45° consistent rotation of the output mode (see Fig. 3d-3, resembling the even HE_21_ mode) in comparison to the initially excited HE_21,odd_ input mode (see Fig. S3a in supplementary information III) was observed for all spectral domains, which can be explained by a twist of the LCF. Assuming a weak ellipticity that is beyond the measurement accuracy of 50 nm in SEM imaging, the induced birefringence makes the fiber slightly polarization-maintaining. This rotation is not visible in case of the rotational symmetric TE_01_ and TM_01_ modes.

### SCG in TE_01_ mode using different fiber core diameters

**Figure 4:**
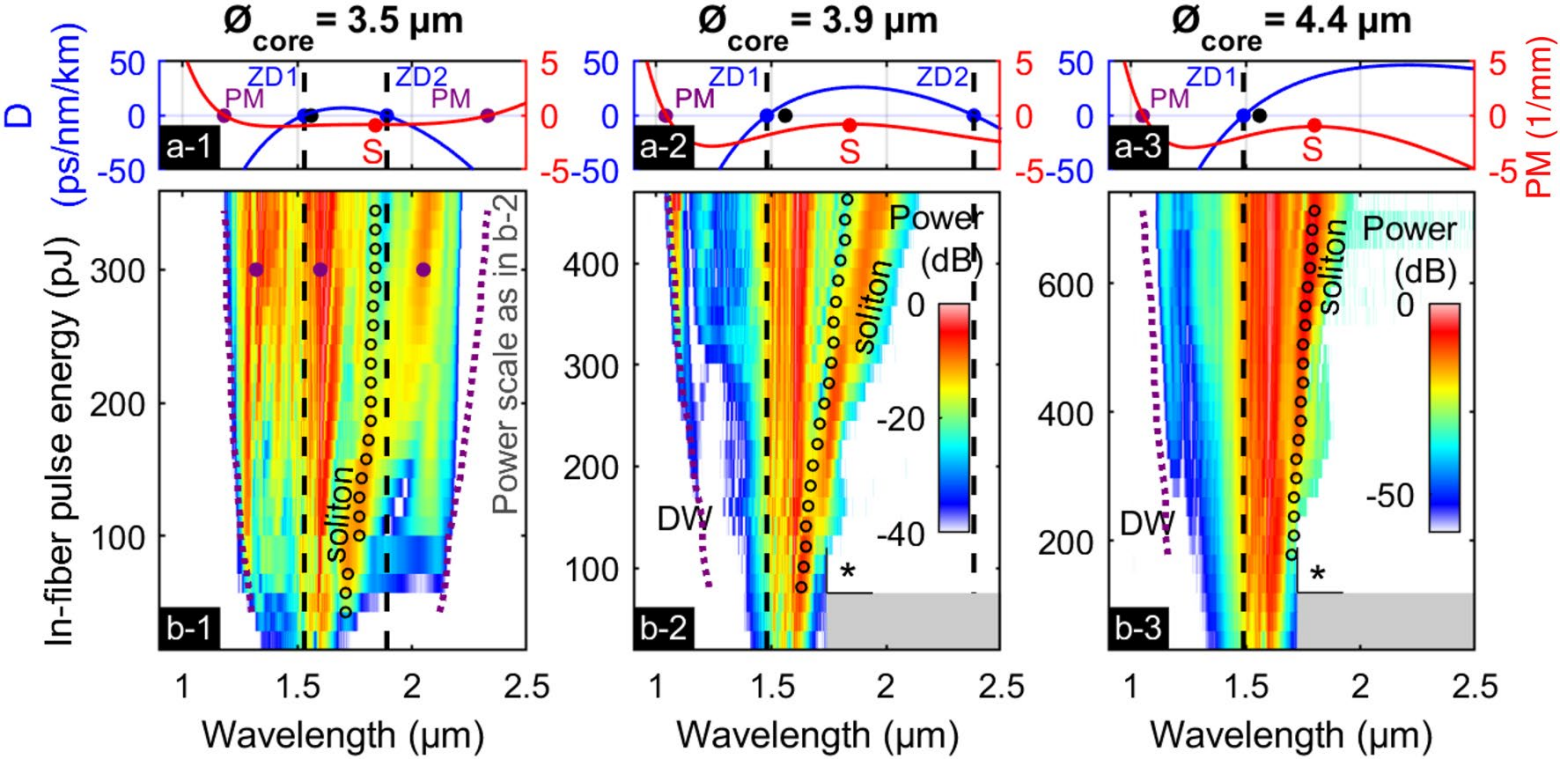
Supercontinuum generation in the TE_01_-mode in three CS_2_-core fibers having different core diameters (Ø_core_ = 3.5 µm (column 1), Ø_core_ = 3.9 µm (column 2), Ø_core_ = 4.4 µm (column 3)). The top row (**a**) shows the group velocity dispersion parameter D (blue, left axis) and phase-mismatch (PM) in 1/mm (red, right axis), while the energy-spectral evolutions are shown in the bottom row (**b)**. The energy-dependent intra-modal phase-matching wavelengths (purple dotted lines) are calculated for the respective soliton wavelengths (black circles). The asterisk (*) marks the start of the spectra with reduced dynamic range, detected with a less sensitive spectrometer. No experimental data is available in the gray shaded regions. The big purple dots in (**b-1**) mark exemplarily the wavelengths matched by degenerated four-wave mixing between a pump at 1.6 µm in the AD regime and two wavelengths in the ND regime.

To reveal the dispersion sensitivity of the HOMs supported in the CS_2_-LCFs, measured energy-spectral evolutions of the TE-excited SCG process are compared for three fibers with different core diameters (3.5 µm, 3.9 µm (as in Fig. 3c-2), and 4.4 µm) in Fig. 4. Measured key parameters as well as calculated dispersion and nonlinear parameters are presented in Table 2.

**Table 2:** Measured key quantities, calculated dispersion, and nonlinear parameters of the investigated fibers, determined at ^a^max. in-fiber energy E_max_, ^b^at onset energy of the respective DW E_DW_, ^c^at λ_P_, and ^d^at 350 pJ.

Ø_core_ [µm]	ε_coupl_ [%]	E_max_ [pJ]	λ_S_^a^ [µm]		E_DW_ [pJ]	λ_DW_^b^ [µm]	λ_ZD1_ [µm]	λ_ZD2_ [µm]	D^c^ [ps/nm/km]	A_eff_^c^ [µm^2^]	γ^c^ [1/W/km]	N^c,d^
3.5	25	360	2.07		43	1.29	1.53	1.89	2.7	10	58	13
3.9	34	452	1.96		169	1.17	1.48	2.38	10.8	12	50	6
4.4	52	714	1.82		268	1.20	1.49	3.07	11.5	14	42	4

The variations in the spectral characteristics of the light generation process correlate well with the dispersive properties of the respective fiber: while the short-wavelength ZDW (λ_ZD1_) remains almost constant for all three LCFs, the ZDW at long wavelengths (λ_ZD2_) substantially red-shifts for increasing core diameters (tuning slope dλ_ZD2_ / dØ_core_ ≈ 1.5 µm/µm). Simultaneously, the measured wavelength λ_S_ of the first fundamental soliton upon fission at maximum energy *E*_max_ decreases with core diameter due to an increase of dispersion *D* (Fig. 4a). For all three fibers the calculated fission lengths at maximum pulse energy is located between 1 cm and 3 cm of propagation distance which is much shorter than the sample lengths used (≈ 9 cm). The measured onset energy (*E*_DW_) of the short-wavelength DW increases strongly with increasing core diameter, while the corresponding wavelength (λ_DW_) decreases. This increase in fission energy results from the reduction of the nonlinear parameter, i.e., from an increasing mode area *A*_eff_, and from the increase of the group velocity dispersion *D*, which simultaneously decreases the fission length. The phase-matching wavelength calculated by Eq. (1) matches well with the position and blue-shift with increasing energy of the experimentally detected short-wavelength dispersive waves.

The fiber with the smallest core (Ø_core_ = 3.5 µm, Fig. 4a-1) reveals another interesting nonlinear dynamics: Here, both ZDWs are close to the pump and the dispersion parameter in the AD regime has a comparably low maximal value (*D*_max_ = 7 ps /nm /km), resulting in a large soliton number *N* = 13 at λ_P_ for a pulse energy of 350 pJ, see Table 2. Figure 4b-1 shows that the red-shift of the soliton is limited by the second ZDW (λ_ZD2_ = 1.89 µm), indicating the boundary to the ND regime where solitons do not exist. Note that the calculated ZDW matches well with the spectral location of the experimentally observed energy drop of the soliton, indicating once more the correctness of the dispersion model. For in-fiber energies up to 200 pJ the split-off soliton (λ_S_ = 1.7 µm) pumps two phase-matched DWs (λ_DW1_ = 1.29 µm, λ_DW2_ = 2.12 µm). For higher in-fiber energies additional spectral components in the normal dispersive domains arise at around 1.33 µm and 2.05 µm, which fulfill energy and momentum conservation of degenerate four-wave mixing with the main peak of the pump at 1.6 µm (purple dots in Fig. 4b-1). Hence, we might observe efficient ultrafast energy transfer to a perfectly phase-matched signal band in the normal dispersive domain, which is seeded by the DW generated earlier^53–55,58,59^. Single-mode simulations solving the nonlinear Schrödinger equation for increasing pulse energy^23^ show a very similar spectral evolution with one major peak in each ND domain left and right of the pump, see supplementary information VI Fig. S7. In case of the two fibers with larger cores (Ø_core_ = 3.9 µm and Ø_core_ = 4.4 µm, Fig. 4b2–3) the soliton does not reach the long-wavelength ZDW and no spectral components are detected at wavelengths longer than λ_S_ due to the low contrast of the used spectrometer (-25 dB for λ > 1.7 µm). On a final note, additional calculations proved that the GVDs of TM_01_ and HE_21_ modes change similarly strong within the chosen core diameter range, whereas the GVD of the fundamental mode remains almost unaffected by these diameter changes.

## Discussion and perspectives

Our study on the first three HOMs proved that CS_2_-core step-index fibers are a suitable platform for SCG in a dispersion landscape with two ZDWs, when exciting HOMs. Even if the long-wavelength DW could only be detected experimentally in case of the smallest core investigated (3.5 µm), the match between phase-matching calculations and nonlinear simulations for the double DW generation verified that two DWs can actually be generated in the capillary-type fiber, when pumped at 1560 nm in the anomalous dispersive regime. This match also proofs that the assumptions of the simulation model (excluding Raman response and laser noise) are valid. The theoretically observed cross-phase induced intermodal DW generation will be investigated in further studies using linear polarized modes, which are more straightforward to demultiplex. This intermodal DW generation in a transversal mode that otherwise has insufficient peak power for soliton fission might soon be used to transfer energy to HOMs, which have promising dispersion designs, but cannot be excited directly, or to nonlinearly switch signals. The measured dumbbell shape of the short-wavelength DWs indicates a non-intuitive polarization behavior suggesting coupling mechanisms that are currently not included in the simulations. To get more insights into these dynamics, our current research aims to perform a similar supercontinuum study in a different waveguide system to prove that this observation is unambiguously a general feature of HOM-SCG. The high sensitivity of the output supercontinua to the excited HOM and to the core diameter leads to a high flexibility regarding dispersion engineering. In contrast to solid core fibers, whose core properties are fixed after the drawing process, the efficiency of dual dispersive wave generation in LCFs can be even further improved via modifying temperature, pressure or liquid mixtures^23,60,61^. Above all, the strong temperature-sensitivity of the HOMs in LCFs represents a very promising property, which allows to change the dispersion on short scales in time and space, and has the potential to surpass the well-known pressure tuning in gas-filled fibers^62^.

## Conclusion

Ultrafast supercontinuum generation of higher-order modes in liquid-core fibers represents a highly attractive scheme for tunable and broadband soliton-based nonlinear frequency conversion. Here, we efficiently excited TM_01_, TE_01_ and HE_21_ modes selectively with spatially and polarization pre-shaped 36 fs-pulses at telecommunication wavelength in CS_2_-silica step-index fibers. The core diameters were adapted carefully, so that the HOMs were pumped at 1560 nm in an anomalous dispersive regime in-between two zero-dispersion wavelengths, defining a dispersion landscape that is typically difficult to established with silica-cladding based step-index fibers. As shown here this allow for dual dispersive wave generation simultaneously into the near- and the short-wave infrared wavelength regions, which can be strongly manipulated by small changes of the dispersion, e.g., induced by changes of the core diameter. The measured energy-spectral evolutions of the individually excited HOMs match with nonlinear simulations and indicate soliton fission and dispersive wave formation as dominant processes of the mode-dependent nonlinear spectral broadening. By spectrally and spatially resolved measurements of the output mode a sophisticated intermodal coupling was found, suggesting non-intuitive intermodal nonlinear dynamics. One interesting feature is intermodal dispersive wave generation, discovered in simulations, which represent the target of future investigations. Overall, our study clearly demonstrates that supercontinuum generation in liquid-core fibers using a low number of higher-order modes represents a highly promising scheme for applications demanding broadband light in a pre-selected polarization state, e.g. multi-modal hyperspectral imaging and next-generation HOM telecommunications. Furthermore, it sets the basis for further experiments manipulating the sensitive dispersion of HOMs by temperature and for emerging photonic machine learning multimode platforms.

## Supplementary Information

Supplementary Information
