# Supplementary material for: Higher-order mode supercontinuum generation in dispersion-engineered liquid-core fibers
Source: Sci Rep. 2021 Mar 5;11:5270. doi: 10.1038/s41598-021-84397-1 (PMC7935952; doi:10.1038/s41598-021-84397-1)
Supplement: Supplementary file 1 — Supplementary Information [file 41598_2021_84397_MOESM1_ESM.pdf]

Supplementary information to:

Higher order mode supercontinuum generation in dispersion engineered liquid core fibers

Ramona Scheibinger,[1] Niklas M. Lüpken,[2] Mario Chemnitz,[3] Kay Schaarschmidt,[1] Jens Kobelke,[1] Carsten Fallnich,[2,4] and Markus A. Schmidt[1,5,*]

[1]Leibniz Institute of Photonic Technology, Albert-Einstein-Str. 9, 07745 Jena, Germany
[2]Institute of Applied Physics, University of Münster, Corrensstraße 2, 48149 Münster, Germany
[3]INRS-EMT, 1650 Boulevard Lionel-Boulet, Varennes, Québec, J3X 1S2, Canada
[4]MESA+ Institute for Nanotechnology, University of Twente, Enschede 7500 AE, The Netherlands
[5]Otto Schott Institute of Material Research, Friedrich Schiller University Jena, Fraunhoferstrasse 6, 07743 Jena, Germany
*markus.schmidt@leibniz-ipht.de

Supplementary information I:
Calculation of modal dispersion and dispersion parameters

The GVD parameter $D = -\lambda / c \times d^2/(d\lambda^2 n_{eff}) = -2\pi c / \lambda^2 \times \beta_2$ is calculated from the effective refractive index n_{eff} of the mode under consideration, which was determined from solving the wave equation of the liquid-core step-index fiber geometry in cylindrical coordinates and is correlated to the group velocity dispersion β_2 [1]. Considering a CS_2-silica step index fiber with a core diameter of $\varnothing_{core}$ = 3.9 µm, the TE_{01} mode has the highest n_{eff} of all HOMs, while the effective refractive index difference Δn_{eff} (TM_{01},HE_{21}) = n_{eff}(TM_{01}) - n_{eff}(HE_{21}) vanishes at 2.1 µm (Fig. S1). The underlying refractive index profile of the CS_2-silca step-index fiber was calculated using the Sellmeier equations of CS_2 and fused silica [2].

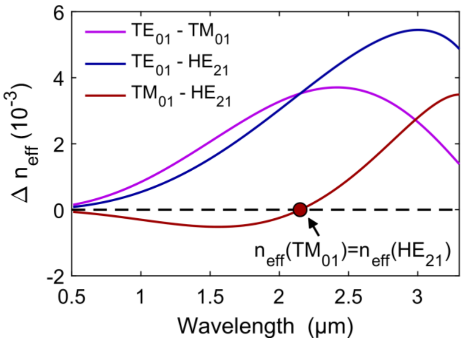

Figure S1. Spectral distribution of the difference of the effective indices of the various modes Δn_{eff} for a CS_2-core fiber with a diameter $\varnothing_{core}$ = 3.9 µm. At 2.1 µm the effective indices of TM_{01} and HE_{21} mode match.

Supplementary information II:
Phase-matching condition in double ZDW dispersion regimes for different soliton wavelengths

Figure S2 demonstrates the influence of the soliton wavelength on both phase-matched DWs in the ND regimes of a system with double ZDWs using the phase-matching equation (Equ.1 of main text), which includes the nonlinear phase of the soliton. A red-shifted soliton wavelength causes both phase-matched DWs to blue-shift and vice versa. In contrast to the short-wavelength DW1, which can be already generated during the initial fission process by a higher-order soliton at pump wavelength (Fig. S2(a)), the wavelength of DW2 obtained by nonlinear simulations (purple line) is phase-matched only when considering the soliton at around 1.85 µm, Fig. S2(c), which corresponds to the split-off fundamental soliton generated after the initial fission process as detected in nonlinear simulations and experiments.

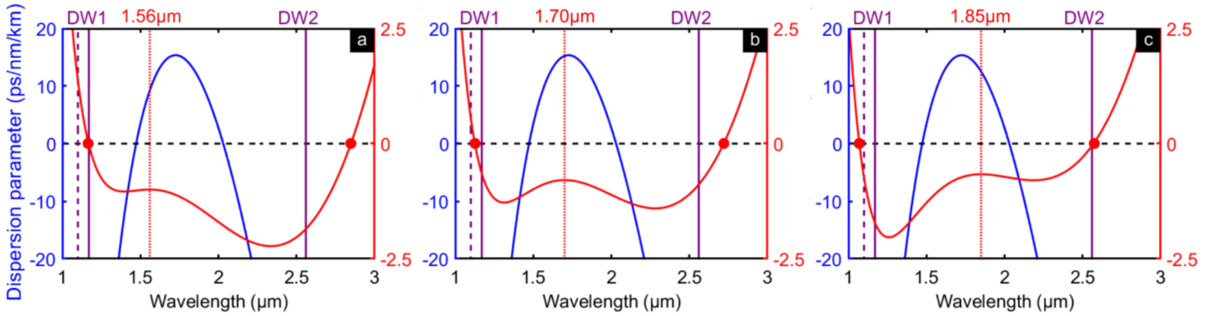

Fig. S2: Dispersive wave phase-matching condition for the TM01 mode in a CS2-core fiber (3.9 µm core diameter, 400 pJ in-fiber pulse energy) for varying soliton wavelengths of (a) 1.56 µm (laser pump wavelength), (b) 1.70 µm and (c) 1.85 µm (fundamental soliton). The red solid line shows the phase-mismatch including the non-linear phase of the soliton (right axis). For comparison, the wavelengths of the dispersive waves obtained by the nonlinear simulations (see Tab. 1) are given as purple vertical lines (exp. results as dotted line). The left axis refers to the GVD parameter (blue solid line).

Supplementary information III:
Stokes parameters of injected beam and theoretical fiber modes

To determine the Stokes parameters S_0, S_1, S_2 and S_3 of the fiber input beam, four intensity images are taken after the initially Gaussian beam is converted to ring-shaped beams with radial/azimuthal polarization by the s-wave plate (and an additional half-waveplate for the HE_{21}-like polarization). Three images are taken behind a linear polarizer at 0°, 45° and 90°, while for the fourth image an additional quarter-wave plate is inserted (polarizer at 45°); both components are removed after characterization [3,4]. The measured spatial distributions of the first three Stokes parameters (S_0, S_1 and S_2) of the input beam (Fig. S3(a)) are in good agreement with the Stokes parameters calculated from the simulated fiber modes (Fig. S3(b)). The last Stokes parameter S_3 characterizing circular polarization does not vanish in experiments as predicted by simulations. This discrepancy is caused by the quarter-wave plate, which is only inserted for the measurement of S_3. The concentric lopes present in all measurements result from diffraction as the beam was slightly bigger than the s-wave plate aperture.

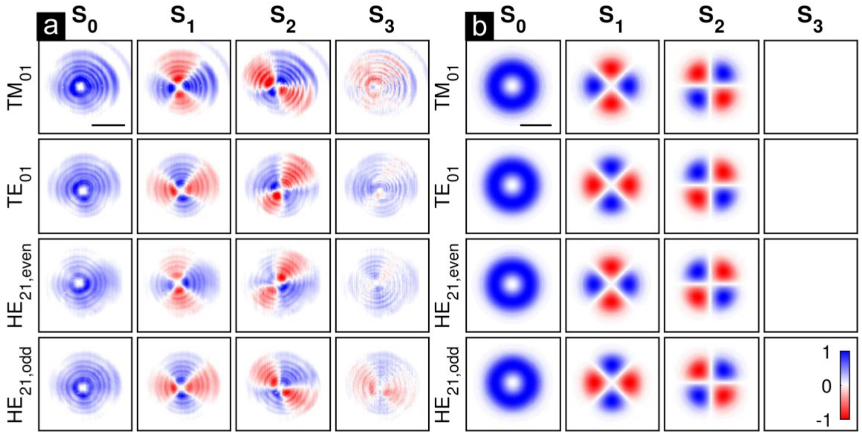

Figure S3. Stokes parameters S_0, S_1, S_2 and S_3 of (a) measured input beams before the coupling lens and (b) simulated higher-order TM_{01}, TE_{01} and HE_{21} modes. The appropriate scale bars are shown in the top left plots of (a) (2 mm) and (b) (2 µm). The color scale in the lower right corner applies for all images.

Supplementary information IV: Overlap calculations and focusing of fields

To estimate the power distribution across the different higher-order modes at the input of the sample, the electric field of the experimental beam E_{exp} is extracted from the Stokes parameter measurements after the s-waveplate. To account for the focusing of the incoupling lens, the fields are numerically focused by geometric refraction on a reference sphere while maintaining the intensity law, as explained in the book *Principles of Nano Optics* [5]. The modal amplitude a_{mode} is a measure of the fraction of power in a particular fiber mode and was calculated by the 2D overlap integral of the cross-product of the electric field vector $\boldsymbol{E}_{exp}$ of the numerically focused beam and the magnetic field vector $\boldsymbol{H}_{mode}$ of the fiber modes taking into account its total power P_{mode} [6]

$$a_{mode} = P_{mode}{}^{-1} \iint_{-\infty}^{\infty} E_{exp}^{focus} \times H_{mode}\, dA. \tag{S1}$$

Supplementary information V: Coherence properties and example evolution calculated by multi-mode nonlinear pulse propagation simulations

In order to quantify the spectral coherence (exemplarily for the HE_{21} mode), 100 output spectra were simulated independently with random shot-noise distributions and an ensemble average was calculated to obtain the first-order coherence according to reference [7]. The average output spectrum (red curve, right axis) and the coherence (blue curve, left axis) are shown in Fig. S4 and reveal a very high spectral coherence.

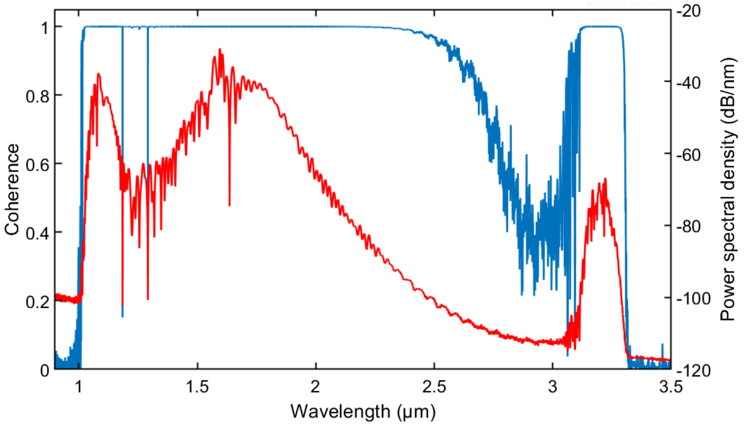

Figure S4. Simulated coherence (blue curve) and averaged output spectrum (red curve) from 100 independent numerical simulations of the HE_{21} mode.

Both, the temporal and spectral evolution as a function of the propagation distance of the TM_{01} mode (Fig. S5), show typical soliton dynamics during SCG. By self-phase modulation in combination with anomalous dispersion the pulse spectrally broadens and temporally shortens in the first millimeter of propagation. The maximum spectral extent is reached at the fission point of about 1 cm. At this point the higher-order soliton decays into fundamental solitons accompanied by dispersive wave generation. After 2 cm of propagation, no significant changes in the spectrum are observed as only dispersive effects occur because there is no significant temporal overlap between the spectral components.

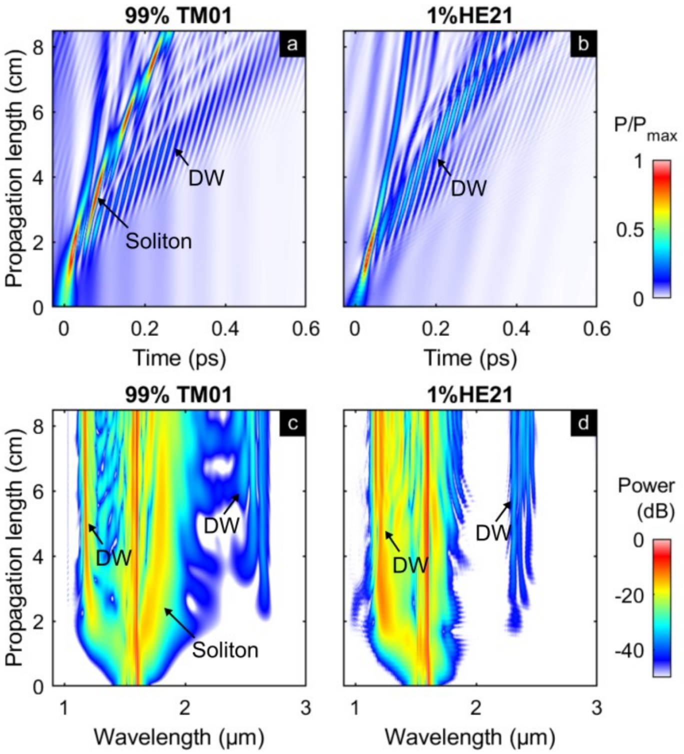

Figure S5. (a-b) Temporal and (c-d) spectral evolution of the TM_{01} mode (99%) and HE_{21} mode (1%) as a function of the propagation distance, excited simultaneously in a CS_2-core fiber with $Ø_{core}$= 3.9 µm and in-fiber pulse energy of 403 pJ. Note that the temporal and spectral fields are normalized independently for both modes to their respective maximum for better visibility of the weak HE_{21} mode.

Figure S6 shows simulated spectra of individual modes excited simultaneously with different modal amplitudes (a) 99 % TM_{01}, 1 % HE_{21}, (b) 96 % TE_{01}, 3 % TM_{01}, 1 % HE_{21} in the CS_2-core fiber ($Ø_{core}$ = 3.9 µm with a total in-fiber energy of 403 pJ. The individual spectra correspond to the spectra shown in Figs. 3b-1 and 3b-2 where the sum of the spectral power densities of all contributing modes is plotted. In Fig. S6 the generation of new frequency components in the weakly excited modes is clearly visible, which is a result of nonlinear intermodal coupling. Calculations of the intra-modal phase-matching between soliton and DWs (triangles in Fig. S6) match well with the simulated spectral positions of both short- and long-wavelength DWs of the strongly excited mode.

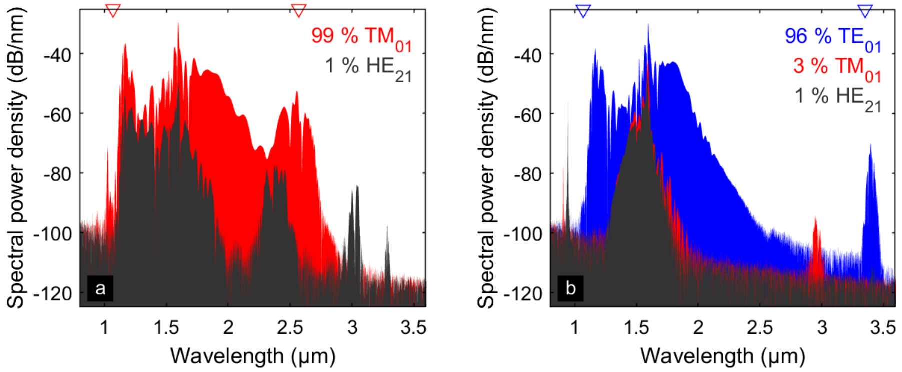

Figure S6. Simulated spectra of strongly and weakly excited higher-order modes for a total in-fiber energy of 403 pJ injected into a CS_2-core fiber with $Ø_{core}$= 3.9 µm. The input power ratios are (a) 99 % TM_{01}, 1 % HE_{21}, (b) 96 % TE_{01}, 3 % TM_{01}, 1 % HE_{21}. The triangles mark the calculated phase-matching wavelengths for the prominent mode.

Supplementary information VI: Single-mode simulation for close ZDWs

Figure S7(b) shows the nonlinear single-mode simulation [2] of the spectral evolution with increasing in-fiber energy for the TE_{01} in a CS_2-core fiber with $Ø_{core}$= 3.5 µm in comparison to the experimentally measured spectral evolution (Fig. S7(a) identical to Fig. 4b-1 in the main text), which shows a good match. In this special system both ZDWs are separated by less than 400 nm (see Tab. 1 in main text and dispersion parameter D).

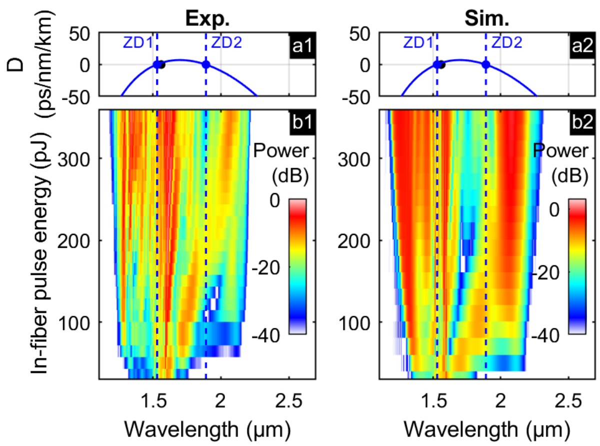

Figure S7. (a) Experimental powerslope for pure TE_{01} mode excitation in a CS_2-core fiber with $Ø_{core}$= 3.5 µm and 8.5 cm length, in comparison to a nonlinear single-mode simulation (for simulation code see [2]) with adapted colormap. The blue dashed lines mark the zero-dispersion wavelength. The central pump wavelength is very close to the short-wavelength ZDW.

References

1. A. W. Snyder and J. D. Love, *Optical Waveguide Theory* (Chapman and Hall, 1983).
2. M. Chemnitz, R. Scheibinger, C. Gaida, M. Gebhardt, F. Stutzki, S. Pumpe, J. Kobelke, A. Tünnermann, J. Limpert, and M. A. Schmidt, "Thermodynamic control of soliton dynamics in liquid-core fibers," Optica **5**, 695–703 (2018).
3. H. G. Berry, G. Gabrielse, and A. E. Livingston, "Measurement of the Stokes parameters of light," Appl. Opt. **16**, 3200–3205 (1977).
4. E. Collett, *Polarized Light in Fiber Optics* (PolaWave Group, 2003).
5. L. Novotny and B. Hecht, *Principles of Nano Optics* (Cambridge University Press, 2006).
6. M. Börner, R. Müller, R. Schieck, and G. Trommer, *Elemente Der Integrierten Optik* (B. G. Teubner, 1990).
7. J. M. Dudley and S. Coen, "Numerical simulations and coherence properties of supercontinuum generation in photonic crystal and tapered optical fibers," IEEE J. Sel. Top. Quantum Electron. **8**, 651–659 (2002).
